# Supplementary material for: Associations of Multimarkers of Metabolic Malnutrition and Inflammation with All-Cause Mortality by Multimorbidity Status
Source: Nutrients. 2025 May 22;17(11):1747. doi: 10.3390/nu17111747 (PMC12157198; doi:10.3390/nu17111747)
Supplement: Supplementary file 1 [file nutrients-17-01747-s001.zip › nutrients-3638898-supplementary.pdf]

Supplementary materials

**Supplementary Material A**	Derivation of the analytic sample
**Supplementary Material B**	STROBE 2007 Statement—Checklist of items that should be included in reports of cohort studies
**Supplementary Material C**	Formulas for computing MVX, IVX, and MMX
**Supplementary Material D**	Summary of missing data
**Supplementary Material E**	Associations of MVX, IVX, and MMX with mortality according to multimorbidity status and age

Supplemental Material A. Derivation of the analytic sample

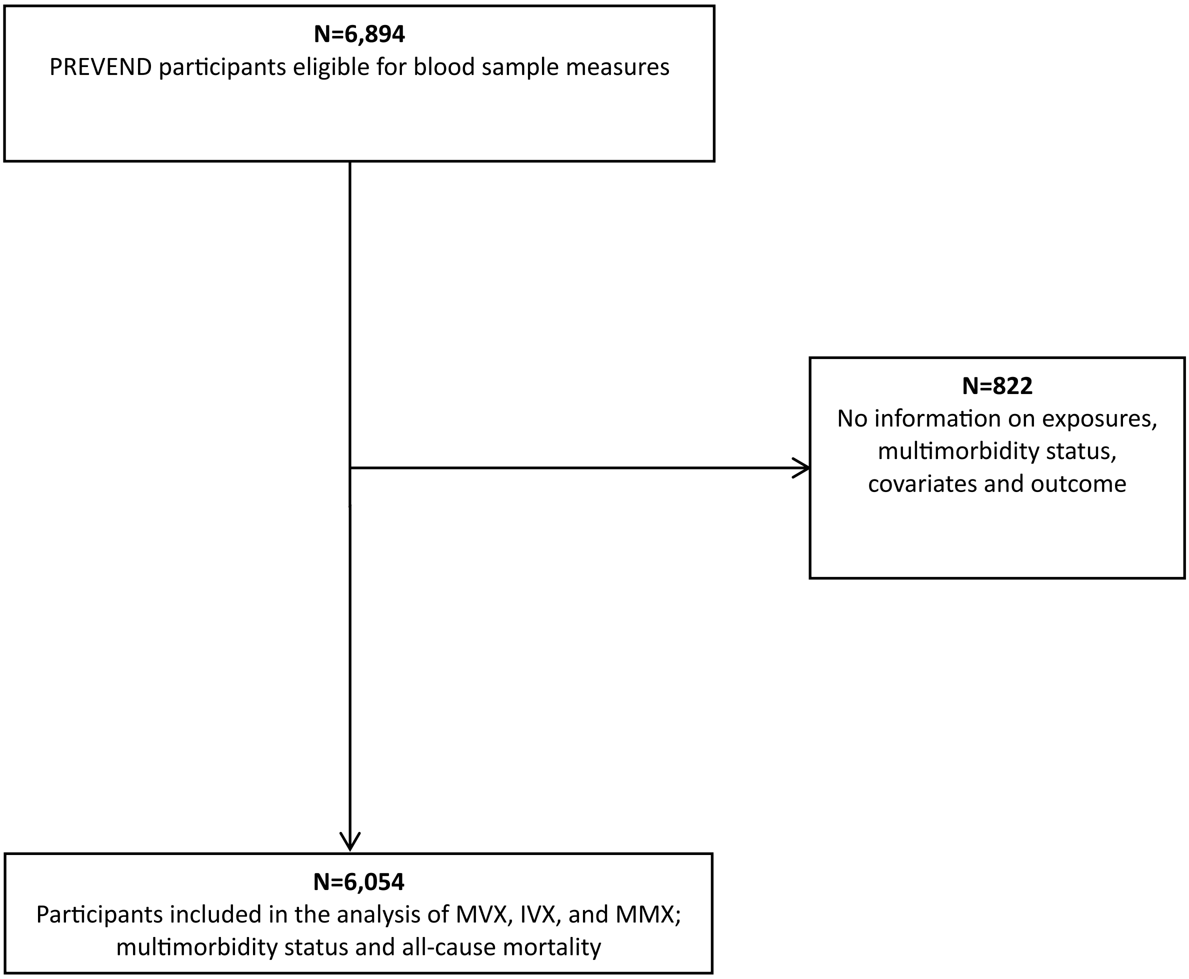

Supplemental Material B. STROBE 2007 Statement—Checklist of items that should be included in reports of cohort studies

**Section/Topic**	**Item #**	**Recommendation**	**Reported on page #**
**Title and abstract**	1	(*a*) Indicate the study's design with a commonly used term in the title or the abstract	Page 1
		(*b*) Provide in the abstract an informative and balanced summary of what was done and what was found	Page 2
**Introduction**			
Background/rationale	2	Explain the scientific background and rationale for the investigation being reported	Page 4
Objectives	3	State specific objectives, including any prespecified hypotheses	Page 4
**Methods**			
Study design	4	Present key elements of study design early in the paper	Study design and population
Setting	5	Describe the setting, locations, and relevant dates, including periods of recruitment, exposure, follow-up, and data collection	Study design and population
Participants	6	(*a*) Give the eligibility criteria, and the sources and methods of selection of participants. Describe methods of follow-up	Study design and population
		(*b*) For matched studies, give matching criteria and number of exposed and unexposed	Not applicable
Variables	7	Clearly define all outcomes, exposures, predictors, potential confounders, and effect modifiers. Give diagnostic criteria, if applicable	Assessment of exposures and other risk markers
Data sources/ measurement	8*	For each variable of interest, give sources of data and details of methods of assessment (measurement). Describe comparability of assessment methods if there is more than one group	Assessment of exposures and other risk markers
Bias	9	Describe any efforts to address potential sources of bias	Statistical analyses
Study size	10	Explain how the study size was arrived at	Statistical analyses
Quantitative variables	11	Explain how quantitative variables were handled in the analyses. If applicable, describe which groupings were chosen and why	Statistical analyses
Statistical methods	12	(*a*) Describe all statistical methods, including those used to control for confounding	Statistical analyses
		(*b*) Describe any methods used to examine subgroups and interactions	Statistical analyses
		(*c*) Explain how missing data were addressed	Not applicable
		(*d*) If applicable, explain how loss to follow-up was addressed	Not applicable

		(*e*) Describe any sensitivity analyses	Statistical analyses
**Results**			
Participants	13*	(a) Report numbers of individuals at each stage of study—eg numbers potentially eligible, examined for eligibility, confirmed eligible, included in the study, completing follow-up, and analysed	Study design and population
		(b) Give reasons for non-participation at each stage	Study design and population
		(c) Consider use of a flow diagram	Study design and population
Descriptive data	14*	(a) Give characteristics of study participants (eg demographic, clinical, social) and information on exposures and potential confounders	Results; Table 1
		(b) Indicate number of participants with missing data for each variable of interest	
		(c) Summarise follow-up time (eg, average and total amount)	Results
Outcome data	15*	Report numbers of outcome events or summary measures over time	Results
Main results	16	(*a*) Give unadjusted estimates and, if applicable, confounder-adjusted estimates and their precision (eg, 95% confidence interval). Make clear which confounders were adjusted for and why they were included	Results; Figure 1; Tables 2-3
		(*b*) Report category boundaries when continuous variables were categorized	Results; Tables 2-3. Figure 1
		(*c*) If relevant, consider translating estimates of relative risk into absolute risk for a meaningful time period	
Other analyses	17	Report other analyses done—eg analyses of subgroups and interactions, and sensitivity analyses	Results
**Discussion**			
Key results	18	Summarise key results with reference to study objectives	Discussion
**Limitations**			
Interpretation	20	Give a cautious overall interpretation of results considering objectives, limitations, multiplicity of analyses, results from similar studies, and other relevant evidence	Discussion
Generalisability	21	Discuss the generalisability (external validity) of the study results	Discussion
**Other information**			
Funding	22	Give the source of funding and the role of the funders for the present study and, if applicable, for the original study on which the present article is based	Title page

Supplemental Material C. Formulas for computing MVX, IVX, and MMX

$IVX = 9 - 0.0027\ GlycA - 0.46079\ sHDLP + 0.0006325\ GlycA \times sHDLP$

$IVX_{min} = 2.0 \Rightarrow$ score = 1

$IVX_{max} = 8.3 \Rightarrow$ score = 100

$MMX = (0.75097\ [4 - 0.02234\ Leu + 0.0000528\ Leu^2]) + (0.55737\ [7 - 0.02895\ Val + 0.0000608\ Val^2]) + (0.00867\ Ile) + (0.65649\ [1 + 0.0025\ Cit + 0.0000167\ Cit^2])$

$MMX_{min} = 1.281 \Rightarrow$ score = 1

$MMX_{max} = 2.0 \Rightarrow$ score = 100

$MVX = 2.72923\ IVX + 11.96062\ \ln MMX - 1.12749\ IVX \times \ln MMX$

$MVX_{min} = 20.3 \Rightarrow$ score = 1

$MVX_{max} = 28.0 \Rightarrow$ score = 100

Cit, citrate; Ile, isoleucine; Leu, leucine; sHDLP, small high-density lipoprotein particle; Val, valine
The subscripts min and max refer to the minimum and maximum values of the corresponding index.

Supplemental Material D. Summary of missing data

Variable	Missing	Total	Percent (%) missing
Age	1	6,894	0.01
Sex	0	6,894	0.00
MVX	336	6,894	4.89
IVX	336	6,894	4.89
MMX	336	6,894	4.89
Valine	204	6,894	2.97
Leucine	204	6,894	2.97
Isoleucine	204	6,894	2.97
Citrate	204	6,894	2.97
GlycA	204	6,894	2.97
Small HDL particles	204	6,894	2.97
Smoking status	86	6,894	1.25
Systolic blood pressure	6	6,894	0.09
Diastolic blood pressure	7	6,894	0.10
Total cholesterol	43	6,894	0.63
High-density lipoprotein cholesterol	204	6,894	2.97
Triglycerides	204	6,894	2.97
Body mass index	0	6,894	0.00
Creatinine	351	6,894	5.10
Cystatin C	343	6,894	4.99
Estimated GFR	352	6,894	5.12
Alcohol consumption	0	6,894	0.00
History of CVD	0	6,894	0.00
History of T2D	0	6,894	0.00
History of CKD	324	6,894	4.71
History of hypertension	51	6,894	0.74
History of cancer	66	6,894	0.96
History of CRD	72	6,894	1.05
All-cause mortality	0	6,894	0.00
Time to event	1	6,894	0.01

CKD, chronic kidney disease; CRD, chronic respiratory disease; CVD, cardiovascular disease; GFR, glomerular filtration rate; HDL, high-density lipoprotein; IVX, inflammation vulnerability index; MMX, metabolic malnutrition index; MVX, metabolic vulnerability index; T2D, type 2 diabetes

Supplemental Material E. Associations of MVX, IVX, and MMX with mortality according to multimorbidity status and age

	Subgroup	Events / Participants	HR (95% CI)	*p*-value*
MVX	**Age <52 years**			
	No disease	46 / 2247	1.32 (0.95, 1.83)	.093
	One disease	24 / 605	1.07 (0.69, 1.66)	.77
	Multimorbidity	13 / 175	1.13 (0.60, 2.12)	.70
	**Age ≥52 years**			
	No disease	140 / 1018	1.28 (1.07, 1.52)	.006
	One disease	243 / 1090	1.25 (1.09, 1.43)	.001
	Multimorbidity	445 / 919	1.30 (1.17, 1.44)	<0.001
IVX	**Age <52 years)**			
	No disease	46 / 2247	1.13 (0.83, 1.56)	.44
	One disease	24 / 605	1.27 (0.84, 1.93)	.26
	Multimorbidity	13 / 175	1.04 (0.57, 1.90)	.91
	**Age ≥52 years**			
	No disease	140 / 1018	1.21 (1.02, 1.45)	.03
	One disease	243 / 1090	1.17 (1.02, 1.34)	.024
	Multimorbidity	445 / 919	1.26 (1.14, 1.39)	<0.001
MMX	**Age <52 years**			
	No disease	46 / 2247	1.45 (1.08, 1.94)	.013
	One disease	24 / 605	0.72 (0.45, 1.14)	.16
	Multimorbidity	13 / 175	1.22 (0.69, 2.17)	.49
	**Age ≥52 years**			
	No disease	140 / 1018	1.22 (1.03, 1.43)	.021
	One disease	243 / 1090	1.21 (1.06, 1.38)	.004
	Multimorbidity	445 / 919	1.15 (1.04, 1.26)	.007

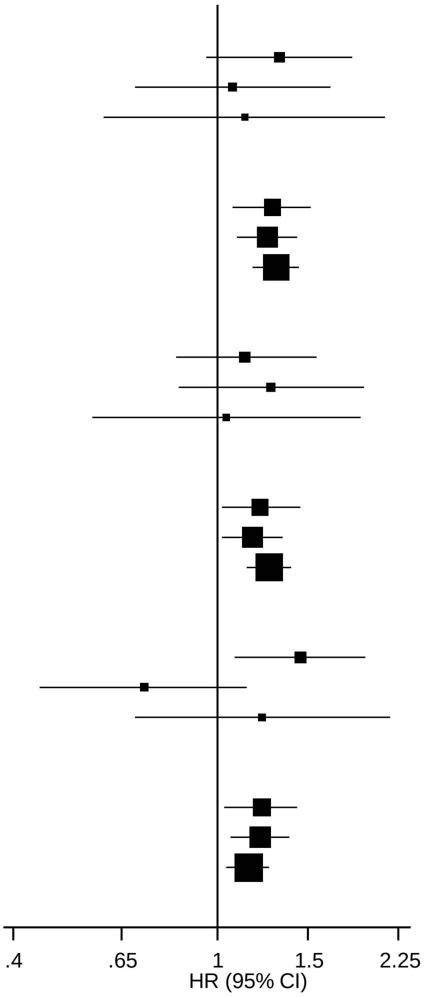

HRs are per 1 standard deviation increase in each exposure
Age cut-off is based on median
CI, confidence interval; HR, hazard ratio; IVX, inflammation vulnerability index; MMX, metabolic malnutrition index; MVX, metabolic vulnerability index
Models were adjusted for age, sex, smoking, alcohol intake, body mass index, total cholesterol, high-density lipoprotein cholesterol, triglycerides, and estimated glomerular filtration rate
